# Integrated microfluidic single-cell immunoblotting chip enables high-throughput isolation, enrichment and direct protein analysis of circulating tumor cells

**DOI:** 10.1038/s41378-021-00342-2

**Published:** 2022-02-02

**Authors:** Aynur Abdulla, Ting Zhang, Shanhe Li, Wenke Guo, Antony R. Warden, Yufang Xin, Nokuzola Maboyi, Jiatao Lou, Haiyang Xie, Xianting Ding

**Affiliations:** 1 Institute for Personalized Medicine, School of Biomedical Engineering, Shanghai Jiao Tong University, Shanghai, 200030 China; 2 Shanghai General Hospital, Shanghai Jiao Tong University, No.85 Wujing Road, Shanghai, 200080 China

**Keywords:** Microfluidics, Biosensors

## Abstract

Effective capture and analysis of a single circulating tumor cell (CTC) is instrumental for early diagnosis and personalized therapy of tumors. However, due to their extremely low abundance and susceptibility to interference from other cells, high-throughput isolation, enrichment, and single-cell-level functional protein analysis of CTCs within one integrated system remains a major challenge. Herein, we present an integrated multifunctional microfluidic system for highly efficient and label-free CTC isolation, CTC enrichment, and single-cell immunoblotting (ieSCI). The ieSCI-chip is a multilayer microfluidic system that combines an inertia force-based cell sorter with a membrane filter for label-free CTC separation and enrichment and a thin layer of a photoactive polyacrylamide gel with microwell arrays at the bottom of the chamber for single-cell immunoblotting. The ieSCI-chip successfully identified a subgroup of apoptosis-negative (Bax-negative) cells, which traditional bulk analysis did not detect, from cisplatin-treated cells. Furthermore, we demonstrated the clinical application of the ieSCI-chip with blood samples from breast cancer patients for personalized CTC epithelial-to-mesenchymal transition (EMT) analysis. The expression level of a tumor cell marker (EpCAM) can be directly determined in isolated CTCs at the single-cell level, and the therapeutic response to anticancer drugs can be simultaneously monitored. Therefore, the ieSCI-chip provides a promising clinical translational tool for clinical drug response monitoring and personalized regimen development.

## Introduction

Detection of circulating tumor cells (CTCs) in the peripheral blood has the potential to be a powerful and noninvasive method for the early diagnosis of cancer invasion and metastasis, evaluation of prognosis, assessment of tumor cell sensitivity to anticancer drugs, and monitoring of therapeutic responses. Technologies have been developed based on the distinctive properties of epithelial CTCs and erythrocytes or leukocytes^1^, such as physical properties (size, weight, or density)^2–6^, flow, and elasticity characteristics^7–10^, and differential expression of biological factors^11,12^. However, simultaneous isolation and downstream molecular characterization of CTCs in the blood of clinical cancer patients remains a major challenge due to the heterogeneity and rarity of CTCs.

Compared to simply enumerating and identifying CTCs, evaluating the expression levels of intracellular target proteins in CTCs is more valuable because it provides unique insight into the underlying biological mechanisms of the specific metastasis and neoplastic heterogeneity of tumors. Owing to the low abundance of many intracellular proteins, measuring the changes in protein expression in single CTCs requires highly sensitive protein analysis technologies. Recently, many novel single-cell immunoassays and related technologies have been developed for the analysis of proteins, for example, DNA-barcoded antibodies and barcode sequencing^13–16^, metal isotope-labeled antibodies for mass cytometry applications^17^, single-molecule enzyme-linked immunosorbent assays^18^, and plasmonic enzyme-linked immunosorbent assays^19^. However, the use of these methods is restricted to the analysis of free and membrane proteins, and complex operational analysis is often required. Integrated systems for CTC isolation, enrichment, and intracellular functional protein profiling are rare.

Recently, single-cell western blotting has shown great potential for multiplex detection of surface, intracellular, and intranuclear proteins at single-cell resolution. Western blotting is usually regarded as the gold standard for protein analysis because the electrophoretic separation step allows a reduction in antibody cross-reactivity, and the immobilization of an antigen at a physical location on the detection membrane is related to molecular size standards. Single-cell western blotting has enabled the monitoring of intracellular protein expression changes in individual CTCs isolated from patients with primary estrogen receptor-positive (ER + ) breast cancer^20^. However, micromanipulator and epifluorescence microscopy equipment are required to isolate and transfer the rare cells into microwells, and rare cells are unavoidably mechanically damaged or physically lost during the delay from blood draw to assay.

**Fig. 1:**
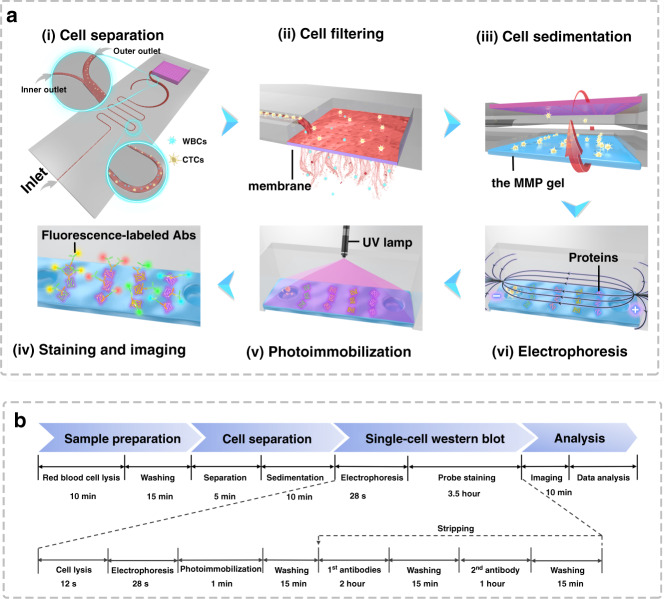
Schematic illustration of the ieSCI-chip pipeline. **a** Overview and schematic illustration of the ieSCI-chip operation procedure: (i) zigzag channel-based label-free and high-efficiency cell sorting to isolate CTCs from background white cells in red blood cell-depleted samples; (ii) membrane-assisted CTC purification and enrichment; (iii) gravity-induced settling of CTCs into microwells on the polyacrylamide gel after inversion of the chip; (iv) chemical lysis of CTCs and electrophoretic separation of the released proteins in the customized electrophoresis chamber; (v) in-gel photoimmobilization of the separated proteins by long-wavelength UV irradiation; (vi) immunoblotting with primary antibodies and fluorescence labeling with secondary antibodies. **b** Timeline of the ieSCI-chip operation procedures

Herein, we combine the advantages of microfluidic label-free sorting and single-cell immunoblotting techniques to develop an integrated isolation, enrichment, and single-cell immunoblotting (ieSCI) microfluidic system. The ieSCI system permits efficient isolation, high enrichment, and direct molecular functional protein characterization of each rare CTC at the single-cell level (Fig. 1). We demonstrate the use of the ieSCI system for rapid isolation and accurate monitoring of cisplatin-stimulated CTCs to quickly differentiate the rare cell population that is resistant to cisplatin treatment. Furthermore, we demonstrate the clinical application of ieSCI to profile the heterogeneity of single-cell EpCAM expression in patient-derived CTCs.

## Results

### Design and operation principle of the ieSCI-chip

The operation principle of the ieSCI-chip is presented in Fig. 1a, b and Supplementary Fig. S1 (online). We exploit a zigzag channel as a label-free and high-efficiency cell sorter to isolate CTCs from blood samples. Then, the chip is inverted to feed the isolated CTCs into the microwells for stippling on the hydrogel for single-cell immunoblotting. The hydrogel serves as a molecular sieving matrix for molecular weight-based protein separation through SDS-polyacrylamide gel electrophoresis (SDS–PAGE) and as a protein immobilization scaffold for in-gel immunoblotting. Herein, we employed a MAPmPyTC-modified polyacrylamide gel (referred to hereafter as the MMP gel)^21^. Previous studies have demonstrated that the photoreactive tetrazole electrophilic addition reaction with a protein as a proximal nucleophile can be completed in a few seconds upon long-wavelength UV irradiation (Fig. 1c)^21^. Compared with traditional benzophenone-based photosensitive gels, the MMP gel has the advantages of excellent electrophoretic separation ability, high protein photocapture efficiency, and low autofluorescence^21^. Next, the chip is transferred to a customized electrophoresis chamber, and RIPA-like lysis and a running buffer is then introduced into the chamber. Afterward, the cells are chemically lysed for 10 s, and the lysates of each single cell are subjected to protein separation via electrophoresis. Proteins are immobilized onto the photoactive gel via long-wavelength UV irradiation for 60 s, which is followed by probing with primary antibodies and fluorescent labeling with secondary antibodies. The intensity of the applied electric field is 40 V/cm. The blots are scanned with a confocal microscope to acquire proteomic data. Here, we designed a microchip electrophoresis section on the chip to accomplish in situ single-cell immunoblotting.

**Fig. 2:**
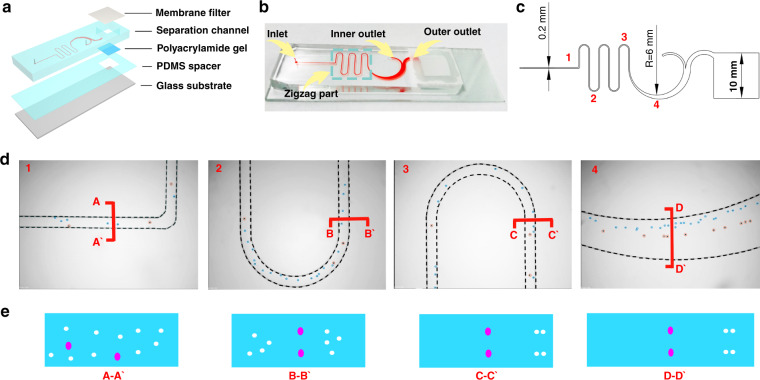
Design and operation principles of the ieSCI-chip. **a** The exploded diagram of the ieSCI-chip. **b** Physical illustration of the ieSCI-chip, which consists of one sample inlet, one inner outlet, and one membrane filter-attached chamber connected to the outer outlet. The separation channel is filled with red dye to make the channel more visible. **c** Corresponding schematic illustration of the ieSCI-chip dimensions. The height of the channel is 80 μm. **d** Distribution of particles (10 and 24 μm to mimic the hydrodynamic behavior of WBCs and MCF-7) at the four marked parts of the channel. The particles in the orange circles are 24 μm, and the particles in the blue circles are 10 μm. **e** Schematic diagram of the particle distribution in the corresponding cross section. The white and pink dots represent 10 and 24 μm particles, respectively

The structure of the ieSCI-chip is shown in Fig. 2a. A physical schematic of the ieSCI-chip is shown in Fig. 2b. Figure 2c shows the specific optimized chip dimensions. The competition between the inertial lift force (*F*_L_) and Dean drag force (*F*_D_) acting on cells inside the zigzag channel gives rise to distinct equilibrium positions for different-sized cells and permits cell separation. In the zigzag portion, cells occupy equilibrium positions in the center of the channel under lower Reynolds number conditions (Supplementary Fig. S2 online). As the Reynolds number increases, large cells (diameter of ~15 μm) distribute near the center of the channel, while small cells flow near the channel walls (Fig. 2d and Supplementary Fig. S3 online). Figure 2e shows the schematic diagram of the particle distribution in the corresponding cross section indicated in Fig. 2d. In the large semicircular channel, when the Reynolds number is low, both the large and small cells keep their equilibrium positions near the center of the channel, without a significant separation distance. With increasing Reynolds number, the small cells flow close to the inner wall and large cells flow near the center of the zigzag channel. When the cells flow through the semicircular channel, *F*_D_ and *F*_C_ become the dominant factors (*a*_p_ ≤ 15 μm), which leads the small cells to move toward the inner channel wall, while the large cells (*a*_*p*_ > 15 μm) move slightly toward the outer wall. When the Reynolds number is higher, the small cells move toward the outer wall, while the large cells move toward the inner wall, making separation difficult.

### Separation efficiency of the zigzag channel portion

**Fig. 3:**
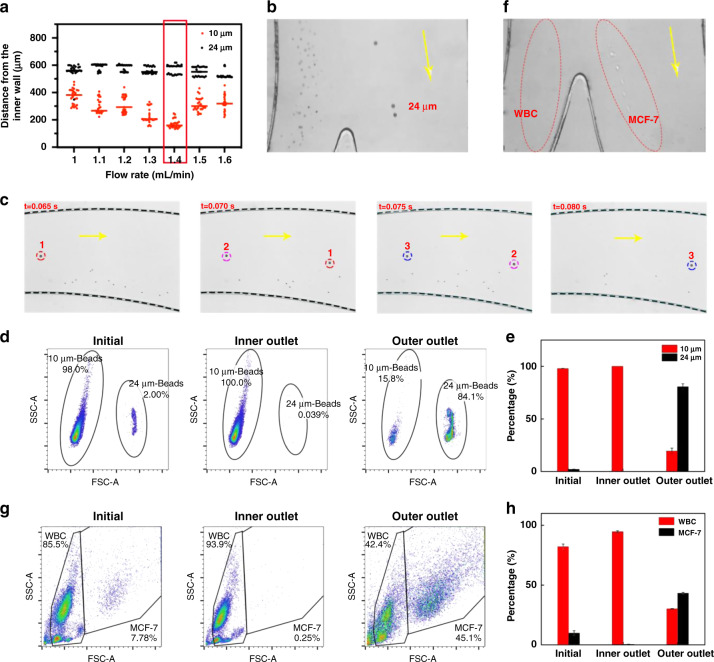
CTC separation performance of the zigzag channel portion alone. **a** Relationship of flow rates and equilibrium positions with respect to the inner channel wall for 10 and 24 μm PS particles. The particle data shown in the figure were acquired from three independent experiments. The flow rate selected for subsequent experiments is shown in the red rectangle. **b** High-speed microscopy images acquired at the bifurcations indicate the separation of the 10 and 24 μm PS particles (a video is available as SI movie 1). **c** Distribution of particles at position 4 at four-time points under a flow rate of 1.4 mL/min, demonstrating that the large particles (24 μm; three representative 24 μm PS particles captured in these time-series images are annotated with numbers and shown in the dotted circles.) flow near the center of the channel, while the small particles (10 μm) flow alongside the wall of the channel. **d** Scatter plots of flow cytometry results showing the proportions of particles (10 and 24 μm) in the initial sample, inner outlet, and outer outlet. The gates were drawn using pure particle samples to discriminate particles of different sizes. **e** Particle size distribution in each outlet. The data were presented as the mean ± SD (SD, standard deviation, *n* = 3) values. **f** High-speed microscopy images acquired at the bifurcations indicate the separation of WBCs and MCF-7 cells (a video is available as SI movie 1). **g** Scatter plots of flow cytometry results showing the compositions of cells collected from the initial sample, inner outlet, and outer outlet. **h** Purity of cells in each outlet. The data were presented as the mean ± SD values (*n* = 6)

To investigate the flow rate and bifurcation of outlets to achieve higher separation efficiency of CTCs, 10 and 24 μm polystyrene particles were used to mimic the hydrodynamic behaviors of WBCs and CTCs, respectively, during the ieSCI-chip optimization process (see Supplementary Materials and Methods). The distances between the 10 μm particles and 24 μm particles under different flow rates were analyzed with ImageJ^©^, as shown in Fig. 3a. Under a low Reynolds number condition (Re = 21.1, corresponding to a flow rate of 1 mL/min), the equilibrium positions of the particles were close to each other. When the Reynolds number was increased to 29.6 (corresponding to a flow rate of 1.4 mL/min), the 10 μm particles and 24 μm particles had the largest separation distance, as shown in Fig. 3b and Supplementary Video S1 (online). When the Reynolds number exceeded 33.9 (corresponding to a flow rate of 1.6 mL/min), the 10 μm particles moved toward the outer wall and were difficult to separate. This effect occurred because *F*_D_ has a dominant effect over *F*_L_ on small particles in the small semicircular channel. The distribution of particles at position 4 (Fig. 2c) at the four-time points under a flow rate of 1.4 mL/min is shown in Fig. 3c, and it demonstrates that after the fluid in the chip becomes steady, the large particles (24 μm) flow near the center of the channel, while the small particles (10 μm) are closer to the inner channel wall and are very stable. The particles that streamed from each outlet under the 1.4 mL/min flow rate were collected and analyzed by flow cytometry to quantify the separation efficiency (Fig. 3d). The purities of the particles in the inner outlet (mainly 10 μm particles) and the outer outlet (mainly 24 μm particles) were 100 and 85%, respectively (Fig. 3e).

A cell sample containing mixed WBCs and MCF-7 cells at a ratio of 10:1 was then injected into the chip at 1.4 mL/min to evaluate the cell separation capacity of the chip. The cells demonstrated equilibrium positions similar to those of the particles (Fig. 3f and Supplementary Video S2 online). Cells were collected from each outlet and analyzed using flow cytometry (Fig. 3g). The purity of MCF-7 cells in the outer outlet was lower than 70% (Fig. 3h), which was lower than that of the polystyrene particles obtained in the above simulation process. This is probably due to the size overlap between CTCs and WBCs, as indicated in the scatter plot in Supplementary Fig. S4 (online).

The ieSCI-chip without a filtering chamber was further tested using the lung cancer cell line A549 and the cervical cancer cell line HeLa to evaluate the ability of the ieSCI-chip without a filtering chamber to separate different kinds of CTCs, and their corresponding cell recovery rates were compared with those of MCF-7 cells (Supplementary Fig. S5 online). The flow rate (as illustrated in Fig. 3) and the chip dimensions (as shown in Fig. 2) for the three cell lines remained the same during the feasibility test. Similar to the procedure described above, A549, HeLa, and MCF-7 cells were stained with DiI and spiked into whole blood at 200 cells/mL. The spiked samples were pretreated as indicated and injected into the ieSCI-chip without a filtering chamber. The cells exiting the CTC outlet were collected and counted under a fluorescence microscope. The cell recovery rate for all cell lines ranged from 87 to 98.5% (Supplementary Fig. S5 online), indicating the desired performance of the separation chip from the perspective of cell recovery.

### Concentration and purification efficiency of the membrane filter-integrated separation chip

**Fig. 4:**
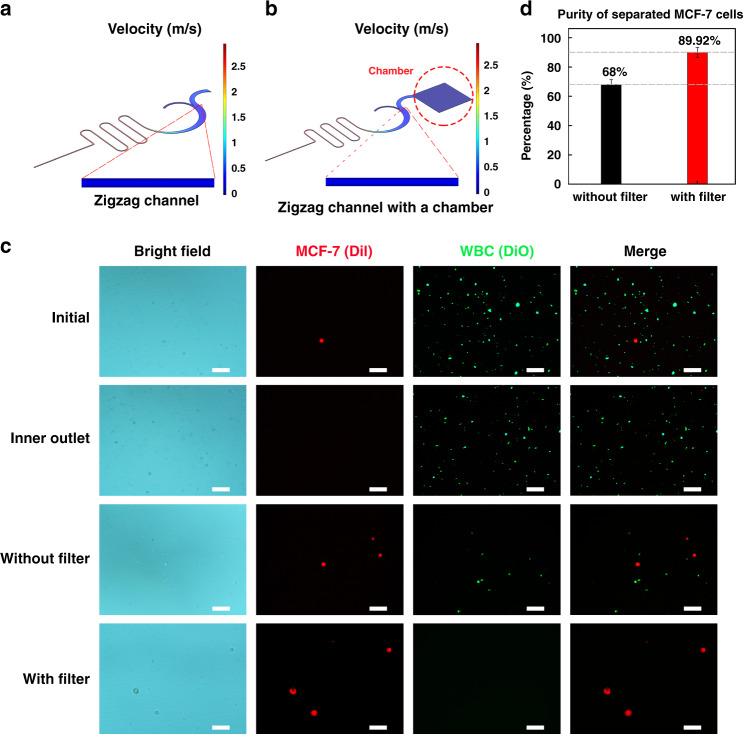
CTC separation, purification, and enrichment performance of the ieSCI-chip. **a** Simulation of the flow rate in the zigzag channel without an additional chamber. **b** Simulation of the flow rate in the zigzag channel with an additional chamber connected. **c** Fluorescence images comparing the initial samples and samples collected from each outlet. MCF-7 cells were stained with DiI (red), and WBCs were stained with DiO (green). The scale bar is 100 μm. **d** Comparison of cell purity with or without the addition of the membrane. The data were presented as the mean ± SD values (*n* = 4)

To reduce residual cell background levels after CTC isolation, we further applied a membrane filter-integrated microfluidics sorter to refine the preliminarily separated CTCs. The chip is placed facedown during the separation process to create a superfluous buffer, and the remaining WBCs are filtered through the membrane. Figure 4a, b shows the flow rate simulation results for the ieSCI-chip without a filtering chamber and the standard ieSCI-chip with a filtering chamber obtained with COMSOL^©^ Multiphysics software. The flow rate distribution inside the zigzag channel remained consistent independent of connection to a chamber. The simulation results indicated that the same flow rate of 1.4 mL/min can be adopted to separate CTCs in the ieSCI-chip. To verify the simulation results and evaluate the separation efficiency of the chip with the filter membrane, DiI-stained MCF-7 cells (red) were spiked into DiO-stained WBCs (green) isolated from healthy donors and suspended in PBS solution, and the mixed cell samples were used to investigate the system’s ability to separate, purify, and enrich CTCs from blood. The inertial focusing of both the CTCs and WBCs was not influenced by the integration of the membrane filter, and the focusing behavior of cells was consistent with that of cells in the ieSCI-chip without a filtering chamber under a 1.4 mL/min flow rate. Corresponding flow fractions exiting the different outlets were collected and imaged using an inverted fluorescence microscope. Figure 4c shows the bright field and fluorescence images of the cell compositions in the initial sample, inner outlet, and chamber with or without a membrane filter. The images clearly indicate that the membrane filter improves the purity of CTCs collected in the chamber. ImageJ^©^ was employed to analyze these images, and the purity of CTCs in the chamber is summarized in Fig. 4d. The purity was significantly increased from 68% to 89.92 ± 3.37% with the addition of the membrane sorter.

Furthermore, the whole blood sample needs to be lysed and diluted before processing, and the volume of the blood sample initially injected into the microfluidic chip is 7.5 mL. With the aid of the membrane filter, the final volume of the isolated CTCs should be the volume of the chamber, which is approximately 0.3 mL. Notably, the CTC enrichment factor can be increased to as high as 25 with the addition of the membrane filter. The high flow rate capability (1.4 mL/min) of the membrane filter-integrated cell sorter allows prompt isolation and enrichment of CTCs within 6 min. Therefore, the developed chip is capable of achieving high-throughput continuous separation and filtration.

### Electrophoresis performance of the integrated ieSCI-chip

**Fig. 5:**
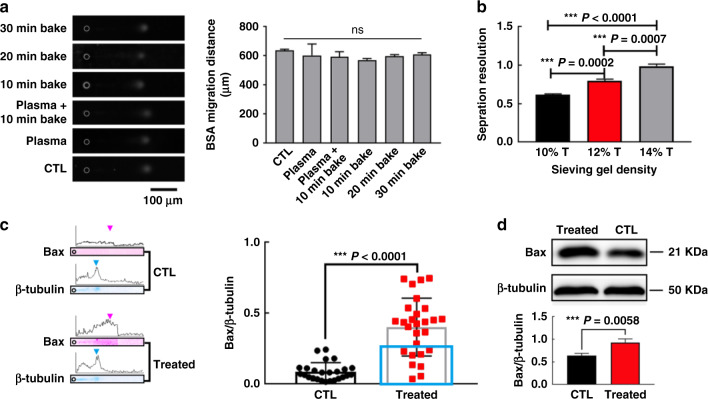
The ieSCI-chip enables monitoring of drug treatment response heterogeneity. **a** Representative fluorescence images (left panel) and quantitative comparison (right panel) of BSA migration behavior in gels subjected to different treatments during chip fabrication. ns indicates nonsignificant. **b** Bar plots show that the separation resolution (SR) improves as the gel pore size is reduced. **c** Left panel: representative images of the single-cell immunoblotting results for an untreated cell and a cisplatin-treated Bax-positive cell. Right panel: the distribution of Bax expression between control and cisplatin-treated cells. The Bax signal in each single cell was normalized to the corresponding β-tubulin signal (control, *n* = 25 cells; cisplatin-treated, *n* = 27 cells). E = 40 V cm^−1^, lysis time = 10 s, electrophoresis time = 25 s, 12% T mPyTC-PA gel). The blue rectangle indicates Bax-negative cisplatin-treated cells. **d** Conventional western blot images (upper panel) and quantitative comparison (lower panel) of Bax and β-tubulin expression in control (CTL) and cisplatin-treated (treated) MCF-7 cells. The data were expressed as the mean ± SD values (*n* = 3)

Next, we investigated the influence of the oxygen plasma and drying treatment during chip fabrication on gel electrophoresis performance. To demonstrate whether the photoactive hydrogel after fabrication treatments is compatible for microfluidic electrophoresis and in situ immunoblotting, FITC-labeled bovine serum albumin (BSA, 66 kDa) and ovarian albumin (OVA, 43 kDa for OVA and 86 kDa for dimeric OVA) were employed. The purified proteins were diluted in PBS and used at a final working concentration of 10 μM. The mPyTC-modified PA (MMP) gel was prepared according to a published protocol^21^, treated with oxygen plasma, and dried in an oven for different durations. Purified proteins were added dropwise onto the MMP gel and were then subjected to electrophoresis. Figure 5a shows fluorescence images of the BSA migration behavior in gels subjected to different MMP treatments during the chip fabrication process. The chip fabrication procedures, including both the oxygen plasma and drying treatments, showed negligible effects on the BSA migration distances, with a constant electrophoresis time of 30 s (Fig. 5a, right panel).

We then investigated suitable gel concentrations for electrophoresis on the ieSCI-chip. BSA dilutions were added to the MMP gel in the chamber as previously described^21^. To achieve high separation performance, we measured the separation resolution^22^ of different MMP gel densities ranging from 10–14% T using a purified fluorescently labeled protein ladder (dimeric OVA, OVA, and a trypsin inhibitor (TI); 21 kDa for TI). Figure 5b displays the separation resolution (SR) based on the TI peak in resolving gels of different densities (% T) at a single time point for ladder migration (*n* = 3). The time point was selected as the time that TI reached a separation distance of 1.5 mm. A higher separation resolution was achieved as the resolving gel density increased. Considering that gels with smaller pore sizes conferred enhanced size discrimination capability but decreased antibody infiltration ability during immunoblotting, a 12% T MMP gel was selected for subsequent analysis.

### ieSCI-chip enables monitoring of drug response heterogeneity

To demonstrate the clinical applicability of the method in individualized therapy, we conducted a drug resistance study by using an ER^+^ breast cancer cell line (MCF-7) to simulate CTCs in ER^+^ breast cancer patients. The estrogen receptor (ER) is expressed in seventy percent of breast cancer patients^23^. Patients with the same type of ER^+^ breast cancer, however, show variable clinical responses to routine chemotherapy treatments. Extraordinary cellular diversity and heterogeneity, including EMT, are recognized as critical factors in chemotherapeutic resistance, tumor metastasis, and reoccurrence^24^. Researchers have demonstrated that aberrant alterations in apoptotic pathways in CTCs are potential mechanisms in chemoresistance^25^.

Based on the photoclick cycloaddition reaction illustrated in Fig. 1, the rapid protein photocapture ability of the photoactive polyacrylamide gel facilitates in situ immunoblotting of isolated CTCs on the same chip. MCF-7 cells were treated for 24 h with cisplatin, which is the major chemotherapeutic agent used to treat breast cancer^26^. The half-maximal inhibitory concentration^27^ of cisplatin, 5.067 μM, was chosen on the basis of the CCK-8 assay results shown in Supplementary Fig. S6 (online). Then, cells were collected and spiked into WBCs isolated from healthy donors to simulate blood samples obtained from cancer patients receiving chemotherapy. The cancer cells were sorted and enriched in the zigzag channel, and proteins were photoimmobilized on the hydrogel after separation by electrophoresis. Finally, the zigzag channel layer was peeled off, and the remaining section of the ieSCI-chip was placed in a customized electrophoresis chamber for subsequent single-CTC immunoblotting. We performed immunoblotting for β-tubulin, an internal control protein, and the apoptosis-associated protein Bax, one of the identified proapoptotic members of the Bcl-2 protein family^28^, in cisplatin-treated CTCs and compared their levels with those in untreated cells (Fig. 5c). Figure 5c shows the distributions of the Bax signal in control and cisplatin-treated MCF-7 cells. Weak or no expression of Bax was detected in the untreated group, which is in accordance with the literature^29^. A significant increase in the Bax expression level was observed in the cisplatin-treated group, indicating a proapoptotic role of Bax in cisplatin-induced apoptosis^26,30^. This discrepancy between the untreated and cisplatin-treated groups was confirmed using conventional western blotting (Fig. 5d). The results indicated that the proposed chip can be successfully applied to isolate CTCs and analyze intracellular proteins in single cancer cells.

Intriguingly, the results obtained with the ieSCI-chip also identified a small subpopulation of Bax-negative cisplatin-treated cells (Fig. 5c, right panel, marked in red box), which suggests heterogeneity among tumor cells. The Bax protein expression levels in single cells represent the apoptosis levels in these individual cells. Increased Bax expression reflects the tumor cytotoxicity of cisplatin. The observation of the small subpopulation of Bax-negative cells indicates that cisplatin treatment exerts a little cytotoxic effect on this subpopulation. This indicates the potential existence of a cisplatin-resistant population, consistent with previous reports indicating that it is easy to induce cisplatin resistance. Notably, conventional western blot analysis fails to identify heterogeneous Bax expression among cell subpopulations under cisplatin stimulation, while the ieSCI-chip provides accurate information for determining whether tumor cells show evidence of resistance to chemotherapeutic drugs.

### The ieSCI-chip enables analysis of EMT in clinical patient-derived CTCs

**Fig. 6:**
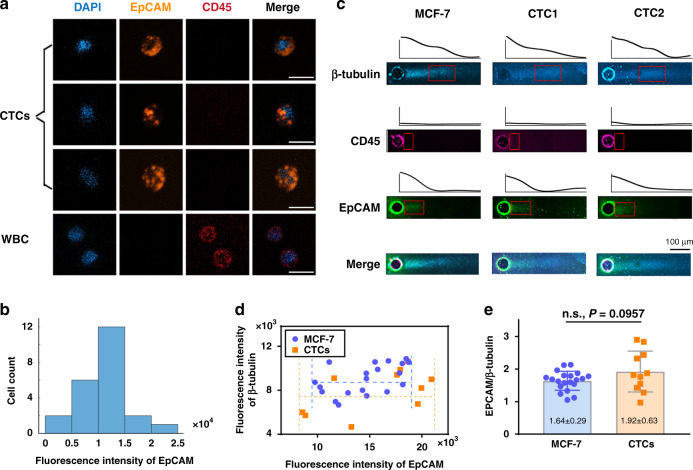
ieSCI-chip enables analysis of EMT in patient-derived CTCs. **a** Representative immunofluorescence images of separated cells from clinical blood samples. Cells were identified as CTCs if the staining pattern was EpCAM^+^ (orange)/CD45− (red)/DAPI^+^ (blue), while cells were identified as WBCs if the staining pattern was EpCAM^-^CD45^+^DAPI^+^. Scale bar, 100 μm. **b** EpCAM expression level distribution via immunofluorescence staining results (*n* = 23 cells. **c** Fluorescence micrographs and intensity plots of MCF-7 cells and representative patient-derived CTCs. Scale bars, 100 μm. Electrophoresis time: 30 s. **d** Expression profiles of individual CTCs and MCF-7 cells based on the expression of β-tubulin and EpCAM via scWB analysis. EpCAM expression was much more diverse in patient-derived CTCs than in MCF cells. The dotted yellow and purple lines indicate the mean ± SD values. **e** Comparison of the EpCAM expression profile between MCF-7 cells and isolated CTCs. The EpCAM expression diversity in CTCs was not detected using bulk statistical analysis. The data were presented as the mean ± SD values. ns indicates nonsignificant

To evaluate the clinical utility of this approach for prognosis prediction, we further measured the expression levels of diagnostic markers in isolated CTCs using clinical blood samples collected from patients with confirmed breast cancer. The demographic characteristics of a representative patient are shown in Table 1. Two tubes of blood (2 mL per tube) were processed, one for CTC enumeration and immunofluorescence staining and the other for performance characterization of the ieSCI-chip. A large number of CTCs (23 per 2 mL of blood) were identified in the patient’s blood sample (Table 1). Figure 6a shows the representative immunofluorescence staining results for separated CTCs (EpCAM^+^CD45^−^DAPI^+^) and WBCs (EpCAM^−^CD45^+^DAPI^+^). The CTCs exhibited distinct EpCAM expression levels (Fig. 6b).

**Table 1:** Representative patient characteristics and the number of EpCAM+/CD45-DAPI+ cells

Sex	Female
Age	45
C-TNM	T4N3M0
Immunohistochemistry	ER-, PR-, ErbB2+++
Cancer type	HER2-positive
Sensitive to adjuvant therapy: TCH	No
CTC (EpCAM^+^CD45^+^DAPI^+^) count per 2 mL	23

The EpCAM expression level is generally believed to indicate the epithelial-mesenchymal transition status and is related to tumor metastasis and progression. Therefore, evaluation of EpCAM expression in individual CTCs is of clinical significance. We thus employed the ieSCI-chip to confirm the EpCAM expression variations in patient-derived CTCs. The blood sample was incubated with red blood cell (RBC) lysis buffer to deplete RBCs, and all the nucleated cells were resuspended in 4 mL 0.9% saline and injected into the ieSCI-chip. Representative immunoblotting results are presented in Fig. 6c. MCF-7 cells were used as a control and for comparison. Figure 6d displays an overview of the EpCAM expression profiles in MCF-7 cells and individual patient-derived CTCs as measured via the ieSCI-chip system. EpCAM expression exhibited slight uniformity across MCF-7 cells (CV = 17.9%, *n* = 20) but much more diversity across patient-derived CTCs (CV = 32.8%, *n* = 11). This might be due to the EMT process, which is a critical process during the metastatic cascade and influences the motility and invasiveness potential of CTCs, or may simply be associated with cellular heterogeneity. However, these differences were not detected when the bulk statistical analysis was used (Fig. 6e). Our results highlighted one major limitation of ignoring or disregarding EpCAM-low or EpCAM-negative CTCs in traditional EpCAM-based CTC detection approaches. The ieSCI-chip is capable of detecting and profiling EpCAM-low/negative CTCs, which may facilitate better prognosis predictions and therapeutic strategy management. In essence, the ieSCI-chip serves as a profiling platform for gauging biological EMT variation among patient-derived CTCs.

## Discussion

CTC characterization is a “liquid biopsy” rather than an Unlike tissue biopsy approach, allowing minimally invasive and real-time biomarker discovery and expression monitoring^31^. However, most of the current clinical applications of CTC detection and monitoring are based on simple enumeration of CTCs^20^. Further characterization, phenotyping, and genotyping of CTCs is needed to provide further accurate functional information for evaluating prognosis and guiding personalized treatment. Previous reports have demonstrated the clinical practice of molecular characterization of CTCs through immunomagnetic separation or other methods to investigate the expression of IGFR1^32^, mammaglobins^33^, or urinary plasminogen activator^34^ in breast cancer patients and of ERG, the androgen receptor, and PTEN60^35^ in prostate cancer patients.

In this study, we developed a seamless integrated microfluidic chip for high-throughput, label-free CTC isolation, CTC enrichment, and single-CTC immunoblotting. The proposed ieSCI-chip has the following advantages: (1) highly efficient CTC isolation—the zigzag channel structure increases the length of the separation channel for CTC separation, promoting the capture of rare cancer cells from background blood cells; (2) label-free CTC isolation—CTCs are isolated in an inertial force-based label-free manner, preventing EMT-induced CTC loss or cell stress responses caused by EpCAM-based immunological methods; (3) damage-free CTC purification for direct analysis—the isolated CTCs are conveniently enriched in the presence of a membrane filter without a reduction in cell viability, and purified cells can be directly used for single-cell immunoblotting; and (4) protein profiling at single-cell resolution—the isolated and enriched CTCs are loaded on-chip into an immunoblotting section for protein profiling at the single-cell level, which enables monitoring of protein expression without the need for additional operations.

Of note, this device integrates CTC isolation, CTC enrichment, and single-cell immunoblotting functions in one microfluidic system. By using the integrated ieSCI-chip, we decreased the requirement for costly equipment and significantly reduced the possibility of sample loss during separation and protein analysis. The ieSCI-Chip provides an effective platform to separate CTCs from patient blood samples and to investigate the potential molecular mechanism of chemotherapeutic irresponsiveness and EMT, enhancing the possibilities for personalized clinical medicine. Dynamic detection of EpCAM expression on CTCs should have prognostic significance in patients with metastatic breast cancer. As a proof-of-concept, we studied EpCAM expression heterogeneity in patient-derived CTCs, and the results were in accordance with previous studies^31,36^. EMT plays an important role in tumor metastasis and presents a challenge for EpCAM-based CTC isolation and detection methods, such as the CellSearch^©^ system^37^. The ieSCI-chip enables efficient CTC capture and characterization without interference from EMT.

Moreover, current mainstream technologies for molecular profiling of CTCs commonly focus on the bulk genome or transcriptome, whereas our chip is designed to characterize protein expression at single-cell resolution. The ieSCI-chip integrates single-cell western blot (scWB) analysis, combining the merits of microfluidics and traditional western blot analysis. Compared with in situ immunofluorescence, whose specificity is limited by antibody cross-reactivity and by limitations on the number of simultaneous detection channels resulting from fluorescence spectrum overlap, scWB can achieve high specificity through SDS–PAGE-based protein separation and high multiplexing ability through multiple rounds of washing and staining. Most notably, we solve the problem of obtaining therapeutic effect predictions by evaluation of biomarkers across individual cells. Additionally, the flexibility in dimension adjustment and flow rate optimization of the zigzag channel facilitates the application of this device not only in breast cancer but also in other diseases, such as lung cancer and cervical cancer (as demonstrated in Supplementary Fig. S4 online). Furthermore, the performance of the ieSCI-chip was investigated with different concentrations of MCF-7 cells spiked with WBCs to better mimic the numbers of CTCs in cancer patients at different stages (as demonstrated in Supplementary Fig. S7 online). The ieSCI-chip could enable earlier detection of tumor recurrence and drug resistance, thus permitting earlier intervention and tailored medicine for personalized therapeutics^38^. However, additional preclinical and clinical research, including well-designed, properly controlled retrospective clinical trials and prospective clinical investigations, is therefore needed to determine whether the benefits of protein profiling of captured rare CTCs at single-cell resolution can better guide treatment and improve prognosis. Moreover, micromanipulation can be utilized to achieve the ideal microwell occupancy and CTC recovery rate for single-cell immunoblotting. Future efforts will be directed at testing the proof-of-concept ieSCI-chip in large-scale clinical practice, investigating single-CTC proteomic data science^39^, and exploring how protein expression heterogeneities in CTCs influence patients’ therapeutic responsiveness and prognosis.

## Material and methods

### Chip design and fabrication

The separation chip used in our work is a zigzag channel. The widths of the inner and outer outlets are 550 and 950 μm, respectively. The outer outlet is connected to a 10 mm × 10 mm chamber. Holes were thoroughly punched in the inlet, outlet, and square chamber (Supplementary Fig. S1b online). The chip consists of five layers. The bottom layer is a microscope slide. A 200 μm PDMS layer with a 10 mm × 10 mm chamber on one side was bonded to the glass by oxygen plasma treatment. Then, the 10 mm × 10 mm chamber was salinized. A PAGE area with microwell arrays was weaved on the 10 mm × 10 mm chamber. The diameter of the microwells is 60 μm. The horizontal distance between two adjacent microwells is 400 μm, and the vertical distance is 2 mm. The glass-bonded PDMS layer with the PAGE area was bonded with the channel layer via oxygen plasma treatment. Before bonding, 30 μL of water was added to the PAGE area to prevent the gel from drying during the process. In the final step, a filter membrane blocked with 5% BSA was pasted above the square chamber of the chip to prevent nonspecific binding of cells during the filtering process. During the experiment, the ieSCI-chip was inverted on an upright microscope (Eclipse Ci-S, Nikon) equipped with a high-speed CCD camera. The chip was washed with DI water to remove dust, and the square chamber was filled with water before sample injection.

### Separation principle of the chip

In this work, we used an inertial focusing zigzag channel consisting of a zigzag portion and a large semicircular channel.

In the zigzag portion, the cells occupy equilibrium positions due to the balance between the inertial lift force (*F*_L_) and Dean drag force (*F*_D_). These two forces are determined by the channel dimensions, fluid velocity, and cell diameter.

Accordingly, *F*_L_ is given as^40^

1 $$F_{\mathrm{L}} = \frac{{\rho Um^2a_p^4}}{{D_h^2}}C_{\mathrm{L}}$$

where ρ is the fluid density, U_m_ is the maximum fluid velocity defined as ${{{\mathrm{U}}}}_{{{\mathrm{m}}}} \approx 1.5{{{\bar{\mathrm U}}}}$ (where ${{{\bar{\mathrm U}}}}$ is the average fluid velocity of the fluid in the channel), a_p_ is the cell diameter, *D*_*h*_ is the hydraulic diameter of the channel defined as ${{{\mathrm{D}}}}_{{{\mathrm{h}}}} = 2{{{\mathrm{wh}}}}/({{{\mathrm{w}}}} + {{{\mathrm{h}}}})$ (where w and h are the channel width and height, respectively), and *C*_L_ is the lift coefficient.

*F*_D_ is caused by the curvature of the channel and is given as

2 $${{{\mathrm{F}}}}_{{{\mathrm{D}}}}\sim 5.4 \,\times\, 10^{ - 4}\pi \mu {{{\mathrm{D}}}}_{{{\mathrm{e}}}}^{1.63}{{{\mathrm{a}}}}_{{{\mathrm{p}}}}$$

where μ represents the viscosity coefficient of the fluid and D_e_ equals the Reynolds number (R_e_) of the channel multiplied by $\sqrt {\frac{{D_h}}{{2R}}}$^41^.

When cells travel through the large semicircular channel, the equilibrium positions of the cells are determined by the force balance between *F*_L_, *F*_D,_ and the centrifugal force (*F*_C_). According to the literature^42^, *F*_C_ is described as

3 $${{{\mathrm{F}}}}_{{{\mathrm{C}}}} = \frac{{\rho _{{{\mathrm{p}}}}\pi {{{\mathrm{a}}}}_{{{\mathrm{p}}}}^3{{{\mathrm{U}}}}^2}}{{6{{{\mathrm{r}}}}}}$$

where *ρ*_*p*_ is the cell density, *U* is the fluid velocity, and *r* is the radius of the equilibrium position of the cells in the semicircular channel.

### Single-cell immunoblotting

Single-cell immunoblotting was performed in accordance with the protocol (Supplementary Fig. S1a online)^43^. Briefly, after cells were settled into the microwells by gravity, they were lysed (∼12 s) in the wells in 1× modified RIPA-like electrophoresis buffer prewarmed to 55 °C, and the proteins were then electrophoresed in the photoactive gel at ∼40 V/cm (∼28 s) in a custom-made electrophoresis chamber. The proteins were immediately photoimmobilized in the gel by a UV-mediated covalent reaction between abstractable hydrogens on the proteins and the mPyTC groups incorporated in the gel matrix (Supplementary Fig. S1a online). During the immunoassay, an antibody in 1× TBST (Tris-buffered saline with Tween 20) with 5% BSA was loaded at the edge of the gel. The gels were probed with primary antibodies for 2 h and with secondary antibodies for 1 h. Between probing steps, the gels were washed two times in 1× TBST for 30 min each on an orbital shaker.

### Fluorescence imaging and image analysis

A Zeiss LSM 880 confocal microscope was employed for image acquisition, and ImageJ^©^ software was used for background subtraction (50 pixels rolling ball radius) and fluorescence quantification of single-cell western blot (scWB) images. Statistical analysis was carried out with GraphPad Prism 8.0.

## Supplementary information

Supplementary information

Supplementary Movie S1

Supplementary Movie S2

## Data Availability

Data are available from the authors upon request.
