# Supplementary material for: Integrated microfluidic single-cell immunoblotting chip enables high-throughput isolation, enrichment and direct protein analysis of circulating tumor cells
Source: Microsyst Nanoeng. 2022 Feb 2;8:13. doi: 10.1038/s41378-021-00342-2 (PMC8807661; doi:10.1038/s41378-021-00342-2)
Supplement: Supplementary file 1 — Supplementary information [file 41378_2021_342_MOESM1_ESM.docx]

**Supplementary Information for**

Integrated microfluidic single-cell immunoblotting chip enables high-throughput isolation, enrichment and direct protein analysis of circulating tumor cells

Aynur Abdulla^a^, Ting Zhang^a^, Shanhe Li^a^, Wenke Guo^a^, Antony R. Warden^a^, Yufang Xin^a^, Nokuzola Maboyi^a^, Jiatao Lou^b^, Haiyang Xie^a,^*, Xianting Ding^a,^*

^a^ Institute for Personalized Medicine, School of Biomedical Engineering, Shanghai Jiao Tong University, Shanghai 200030, China

^b^ Department of Laboratory Medicine, Shanghai Chest Hospital, Shanghai Jiao Tong University, No. 241 Huaihai West Road, Shanghai 200030, China

^*^ Corresponding author. E-mail: hyxie@sjtu.edu.cn and dingxianting@sjtu.edu.cn., Tel: +8617521003308 and +8615601752012, Fax: 86-21-62932274

**Supplementary Materials and Methods**

**Reagents.** TWEEN-20 (P1379), Triton X-100 (T8787), and ammonium persulfate (APS, A3678) were purchased from Sigma-Aldrich. Tetramethylethylenediamine (TEMED, TB0508), acrylamide/bis-acrylamide 30% solution (29:1) (B546017), 10x TBST buffer and other conventional western blot associated buffer were purchased from Sangon Biotech. Deionized water (18.2 MΩ) was obtained with an ultrapure water system from Millipore. N-(3-methacrylamidopropyl)-2-(1-methyl-1H-pyrrol-2-yl)-2H-tetrazole-5-carboxamide (MAP-mPyTC) was synthesized in house as reported in our previous work (27).

**Antibody and protein standards.** Antibodies employed for CTC characterization include rabbit anti-β-tubulin (605102, BioLegend), rabbit anti Bax (ab32503, Abcam), Donkey anti rabbit Alexa-Fluor 647-labeled secondary antibodies (733-605-152, Jackson), and Donkey anti mouse Alexa-Fluor 555-labeled secondary antibodies (ab150106, Abcam). Peroxidase-Conjugated Goat/Rabbit/Mouse Anti-Rabbit IgG (H+L) (33101ES60) was purchased from Yeasen Biotech. Protein standards include trypsin inhibitor (TI), 20.1 kDa; ovalbumin (OVA), 43 kDa; bovine serum albumin (BSA), 66 kDa; OVA dimer, 86 kDa, all purchased from Sangon Biotech.

**Polystyrene particle samples preparation.** To build the ieSCI-chip, we first characterized the separation performance of the chip with polystyrene (PS) particles (Shanghai Biochemical). We evaluated the performance of the microfluidic chip by using particles sized 10 μm and 24 μm to mimic the hydrodynamic behavior of white blood cells (WBC) and MCF-7. The concentrations of the 10 μm and 24 μm particles were set as 5×10^5^/mL and 1×10^4^/mL, respectively. The particles were suspended in DI water with 1% w/v tween-20 to prevent particle agglomeration.

**Cell culture and cisplatin induced cytotoxicity.** A breast cancer cell line (MCF-7, ATCC, U.S.A.) was used in this study to evaluate the hydrodynamic behavior of CTCs in the chip. Cells were cultured in high-glucose DMEM (Thermo Scientific, USA) supplemented with 10% FBS and 1% penicillin–streptomycin at 37°C, in 5% CO_2_. Cisplatin induced cytotoxicity was measured using a cell counting kit-8 (CCK8, DOJINDO, Japan). Briefly, a 100 μL cell suspension (10^4^ cells/well) was seeded and pre-incubated for 24 h then treated with cisplatin (with a final concentration ranging from 0.5 to 128 μM, diluted by complete culture media) for another 24 h. The existing media was removed and replaced with CCK8 working solution, then incubated for 1 h at 37°C for the cytotoxicity assay. Cell viability was determined by measuring the absorbance at 450 nm via a microplate reader (Synergy HT, BioTek).

**Red blood cell lysis** Human whole blood samples were collected from consenting non-cancer patients at the Shanghai Chest Hospital in Shanghai, China. For each subject, 2 mL blood was collected in EDTA-K2 anticoagulant blood collection tube and stored in 4℃. Each tube of blood was lysed with red blood cells lysis buffer (Beijing Solarbio Science and Technology) at a ratio of 1 mL blood to 3 mL lysis buffer at room temperature on a shaking platform for 10 min. Then, cells were collected by centrifugation at 1000 rpm for 5 min at room temperature and washed with 0.9% saline at least 3 times. The deposited cells were suspended with 0.9% saline.

**Fluorescence staining.** Fluorescent staining was performed to facilitate better distinction between WBCs and MCF-7 cells in the chamber. DiI (DiIC18 (3)) and DiO (DiOC18 (3)) were commonly used as cell membrane fluorescence dyes. In this work, DiO was used to stain WBCs, while DiI was used to stain MCF-7. A proportion of WBCs and MCF-7 cells were harvested and separately diluted in DiO and DiI with a cell density of 1 × 10^6^ /mL. Cells were incubated for 30 min in a humidified atmosphere at 37°C containing 5% (v/v) CO_2_. After incubation, cells were centrifuged at 1000 rpm for 5 min. Supernatant liquid was removed and the cells were washed thrice with prewarmed 37°C DMEM.

**Immunofluorescence staining.** For blood collected from clinical breast cancer patients, separated cells from the zig-zag chip were identified by immunofluorescence staining for cell identification and cell counting. The cells collected were sedimented in wells in a Poly-D-lysine (PDL) treated 96-well plate. After 1-hour sedimentation, cells were washed three times by PBS and fixed with 4% Paraformaldehyde for 10 minutes at room temperature followed by washing with PBS thrice. Then cells were blocked with 3% bovine serum albumin (BSA, Miltenyi Biotec, San Diego, CA) at room temperature for 1 hour followed by PBS washing thrice. After blocking, cells were immunostained with mouse antibody anti-human EpCAM (Abcam, USA). Appropriately matched secondary Alexa Fluor 488- conjugated antibodies (BioLegend, USA) were used to identify CTCs. Then, cells were immunostained with APC-conjugated anti-CD45 (BioLegend, USA) to exclude false positives cells. Nucleus were stained with 4′,6- diamidino-2-phenylindole (DAPI; Life Technologies, Carlsbad, CA). After immunofluorescence staining, cells were washed with PBS for image acquisition with a fluorescence microscope (Zeiss LSM 880, Germany).

**Flow cytometry.** PS particles and cells collected from each outlet of separation chips were placed into separate tubes and analyzed with flow cytometry (Canto II, BD Biosciences). The FlowJo software (Treestar) was used for data analysis. All detections were performed in triplicate.

**Conventional western blot.** Conventional WB was performed at room temperature per protocol (44). Briefly, whole cell protein was extracted using RIPA lysis buffer and fractionated with SDS-PAGE. After electrophoresis, the proteins were electrotransferred to polyvinylidene difluoride (PVDF) membranes, blotted with appropriate antibodies, and developed by SuperSignal-enhanced chemiluminescent substrate solution (Pierce Chemical). The band signals were visualized with the Tanon-5200S Chemiluminescent Imaging System (Tanon) and quantified with ImageJ (NIH).

**Synthesis of MMP gel photosensitizing reagent**. We first synthesized 2-(1-methyl-1H-pyrrol-2-yl)-2H-tetrazole-5-carboxylic acid with N-(3-aminopropyl) methacrylamide to create a functional photo-capture product, N-(3-methacrylamidopropyl)-2-(1-methyl-1H-pyrrol-2-yl)-2H-tetrazole-5-carboxamide (MAP-mPyTC) through EDCl/HOBt catalyzed amide condensation reaction. Copolymerizing the synthesized MAP-mPyTC with acrylamide and N, N’-methylenebisacrylamide formed the mPyTC-modified polyacrylamide (MMP) gel. The synthesis procedures are as follows:

Ethyl 2-(1-methyl-1H-pyrrol-2-yl)-2H-tetrazole-5-carboxylate synthesis. To a solution of methyl-1H-pyrrol (5 g, 12.5 mmol) in trifluoroethanol (30 mL) was added PhI(OAc)2 (20 g, 62.5 mmol) at -40 °C. The mixture was stirred at -40 °C under N2 for 2 h, then concentrated to give the product as black oil (20 g). The residue was dissolved in DCM, then ethyl 2H-tetrazole-5-carboxylate (3 g, 21.6 mmol), Cu(OTf)2 (3.1 g, 8.6 mmol), and TEA (16 ml, 108 mmol) were added. The mixture was stirred at room tem-perature under N2 for 24h, then washed with saturated NH4Cl, brine, dried over anhydrous Na2SO4, filtered, and concentrated. The residue was purified by silica gel flash chromatography (PE: EA = 5:1) to afford Ethyl 2-(1-methyl-1H-pyrrol-2-yl)-2H-tetrazole-5-carboxylate as a brown oil. (450 mg, 9.6% yield).

2-(1-methyl-1H-pyrrol-2-yl)-2H-tetrazole-5-carboxylic acid synthesis. To a solution of (Ethyl 2-(1-methyl-1H-pyrrol-2-yl)-2H-tetrazole-5-carboxylate (450 mg, 2 mmol) in 1:1 MeOH/H2O (20 ml) was added LiOH (850 mg, 10 mmol) at 0 °C. The mixture was stirred at room temperature for 2h. Then 2 N HCl was added at 0 °C to adjust pH to 4~5, and the mixture was extracted with EtOAc. the combined organic layer was washed with brine, dried over anhydrous Na2SO4, filtered, and concentrated to afford the 2-(1-methyl-1H-pyrrol-2-yl)-2H-tetrazole-5-carboxylic acid as a brown solid (370mg, 94% yield).

N-(3-methacrylamidopropyl)-2-(1-methyl-1H-pyrrol-2-yl)-2H-tetrazole-5-carboxamide synthesis. To a solution of N-(3-aminoproipyl)methacrylamide (370 mg, 1.67 mmol) in THF (20 ml) was added EDCI·HCl (650 mg,3.34 mmol), HOBt (220 mg, 1.67 mmol) at 0 °C for 30min; then 2-(1-methyl-1H-pyrrol-2-yl)-2H-tetrazole-5-carboxylic acid (300 mg, 1.67 mmol), TEA (850 mg, 8.35 mmol) were added. The mixture was stirred and refluxed overnight. Then the mixture was purified by prep-HPLC to afford N-(3-methacrylamidopropyl)-2-(1-methyl-1H-pyrrol-2-yl)-2H-tetrazole-5-carboxamide as a white solid (41 mg, 6.7 % yield).

**Supporting Movies**

**SI movie 1.** Polystyrene particles sized 10 μm and 24 μm, flowing in the zigzag channel near outlets at 1.4 mL/min flow rate. (AVI)

**SI movie 2.** RBCs depleted blood spiked with MCF-7 cells flowing in the zigzag channel near outlets at 1.4 mL/min flow rate. (AVI)

**Supplementary Figures**

**
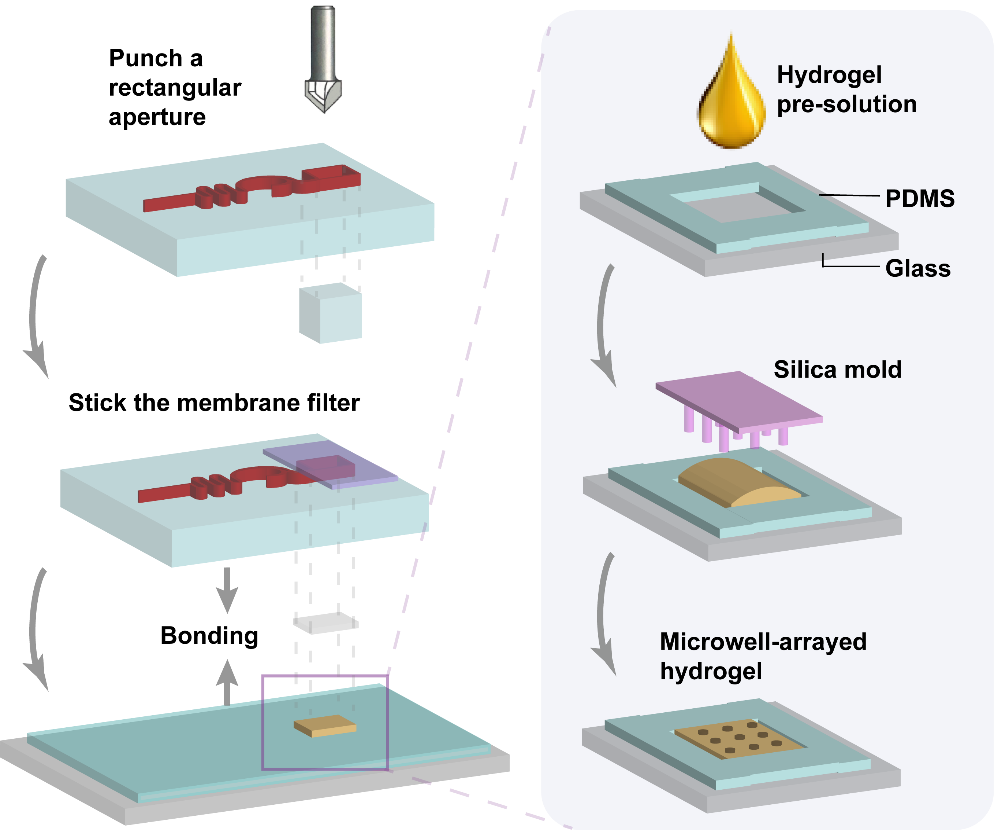
**

**Figure S1.** Workflow of the ieSCI-chip fabrication procedure.


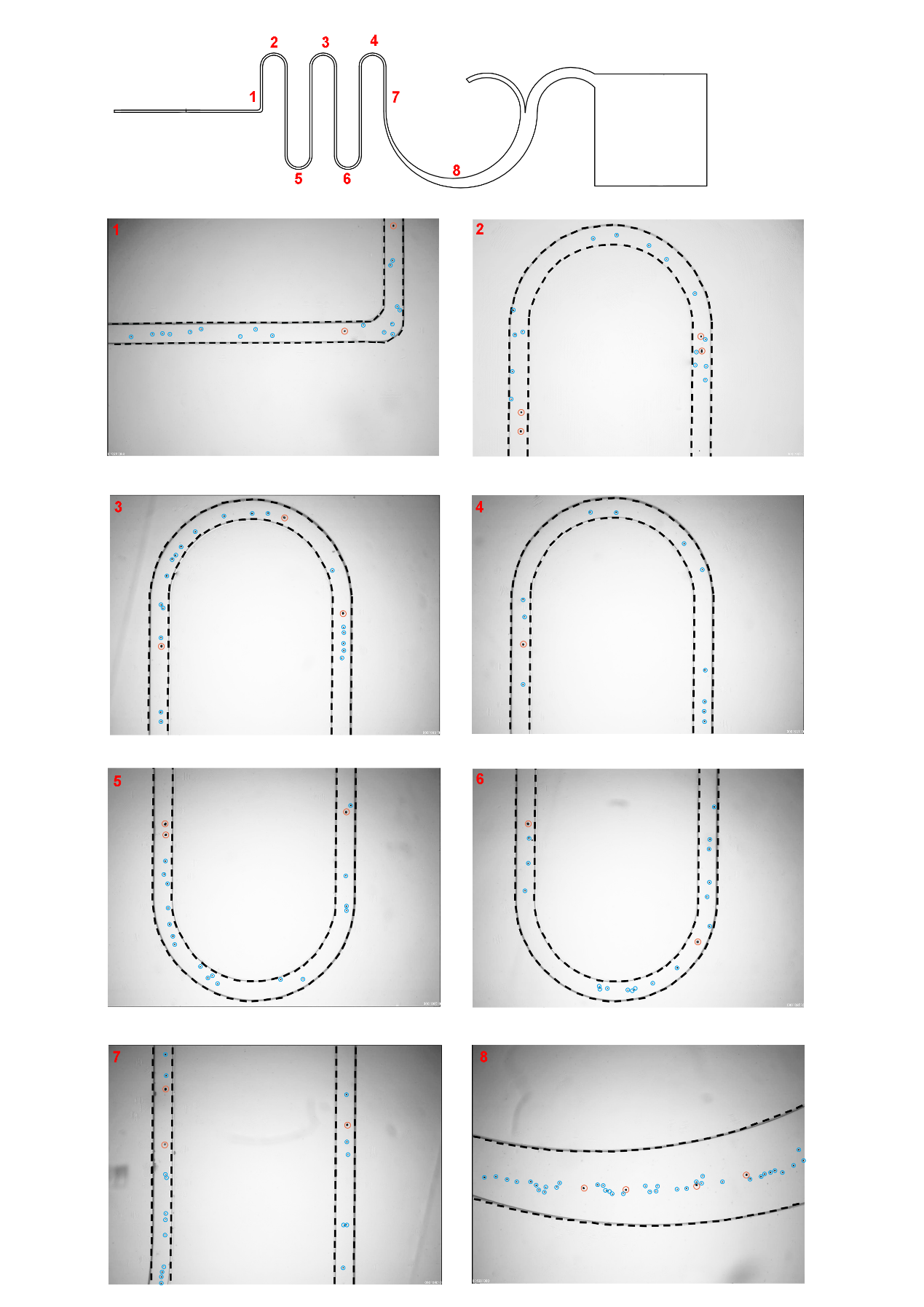


**Figure S2**. Distribution of particles (10 μm and 24 μm to mimic the hydrodynamic behavior of WBC and MCF-7) at the eight marked parts of the channel at a flow rate of 1 mL/min.

**
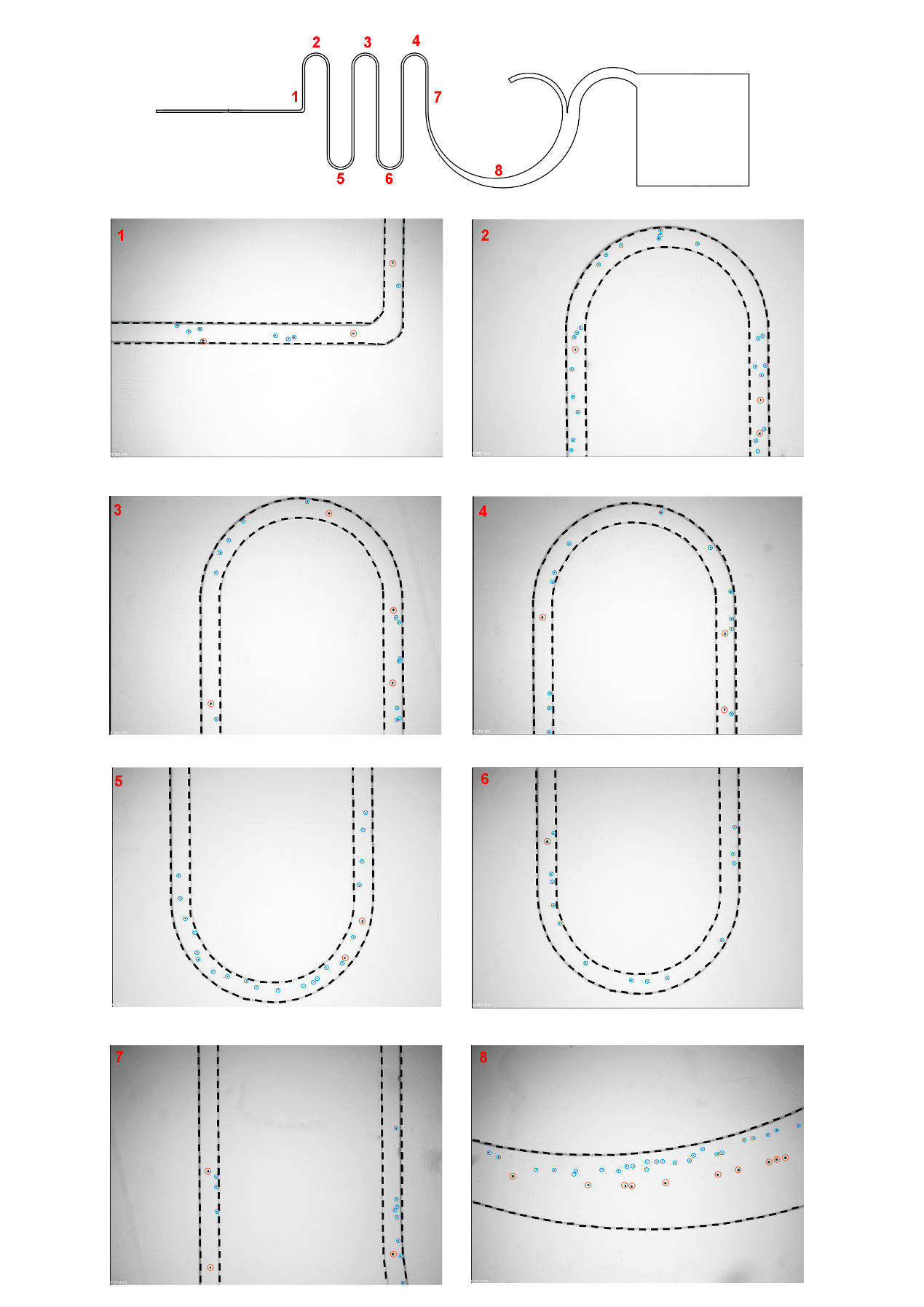
**

**Figure S3**. Distribution of particles (10 μm and 24 μm to mimic the hydrodynamic behavior of WBC and MCF-7) at the eight marked parts of the channel at a flow rate of 1.4 mL/min.

**
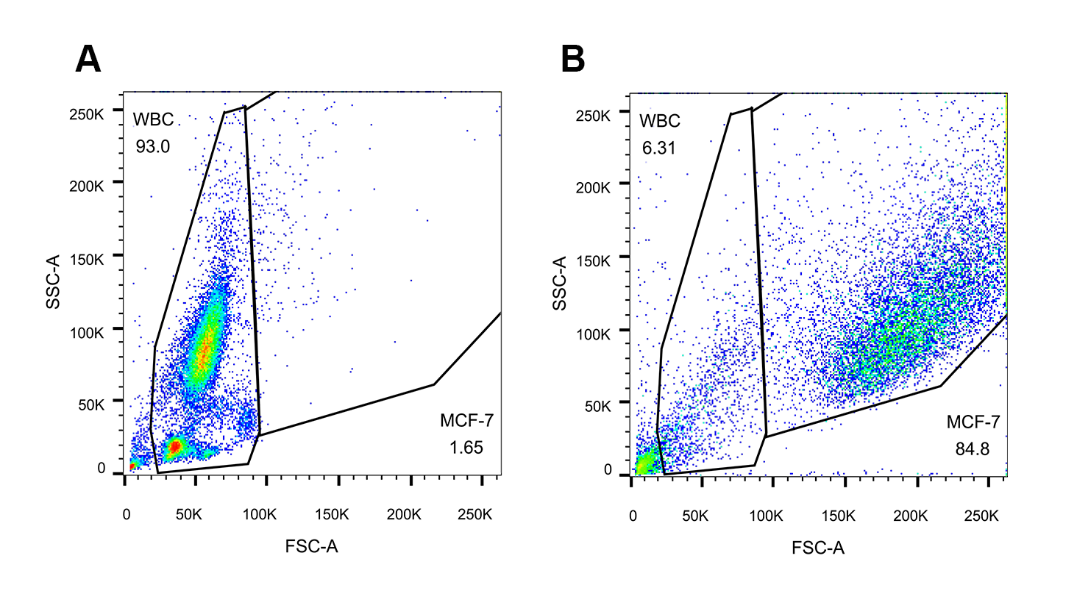
**

**Figure S4.** Scatter plots of flow cytometry results showing the size distribution of WBCs (A) and pure MCF-7 cells (B). These dot plots are provided as controls for gate drawing to discriminate between cell sizes.


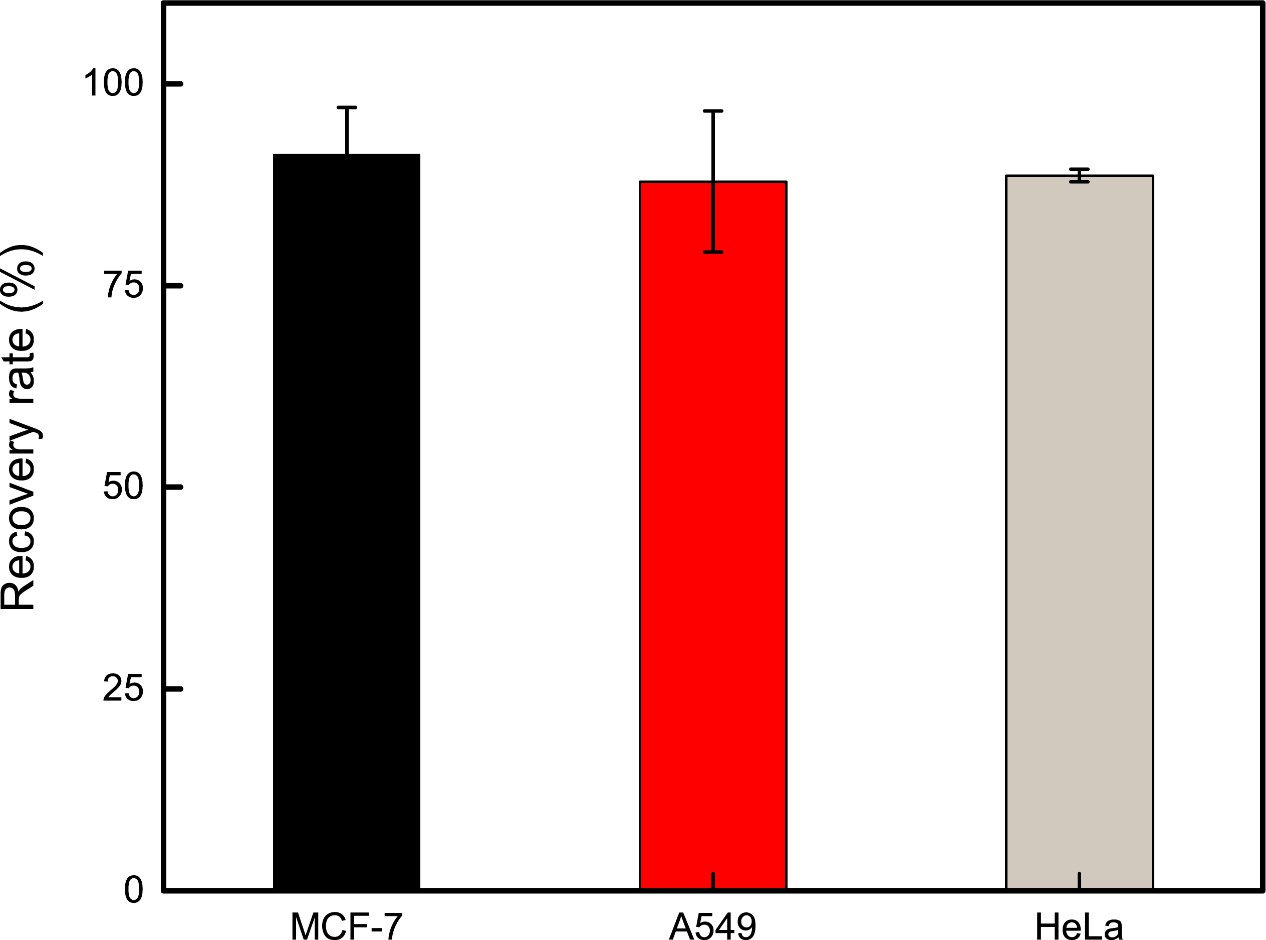


**Figure S5.** Cell recovery rates of MCF-7, A549, and HeLa cell lines.


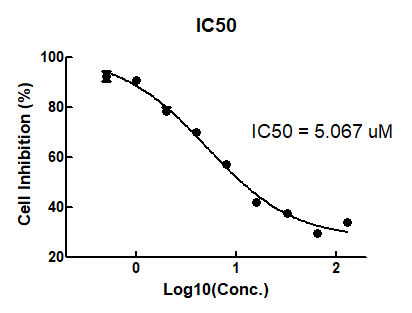


**Figure S6.** Dose-toxicity curve for cisplatin on MCF-7 cells. X axis represents the log value of different concentrations. Values of y axis are expressed as the percentage of cell viability to the control (proliferative activity of control was set as 100%). Data is presented as mean ± SD, where n = 6, each with triplicate samples.


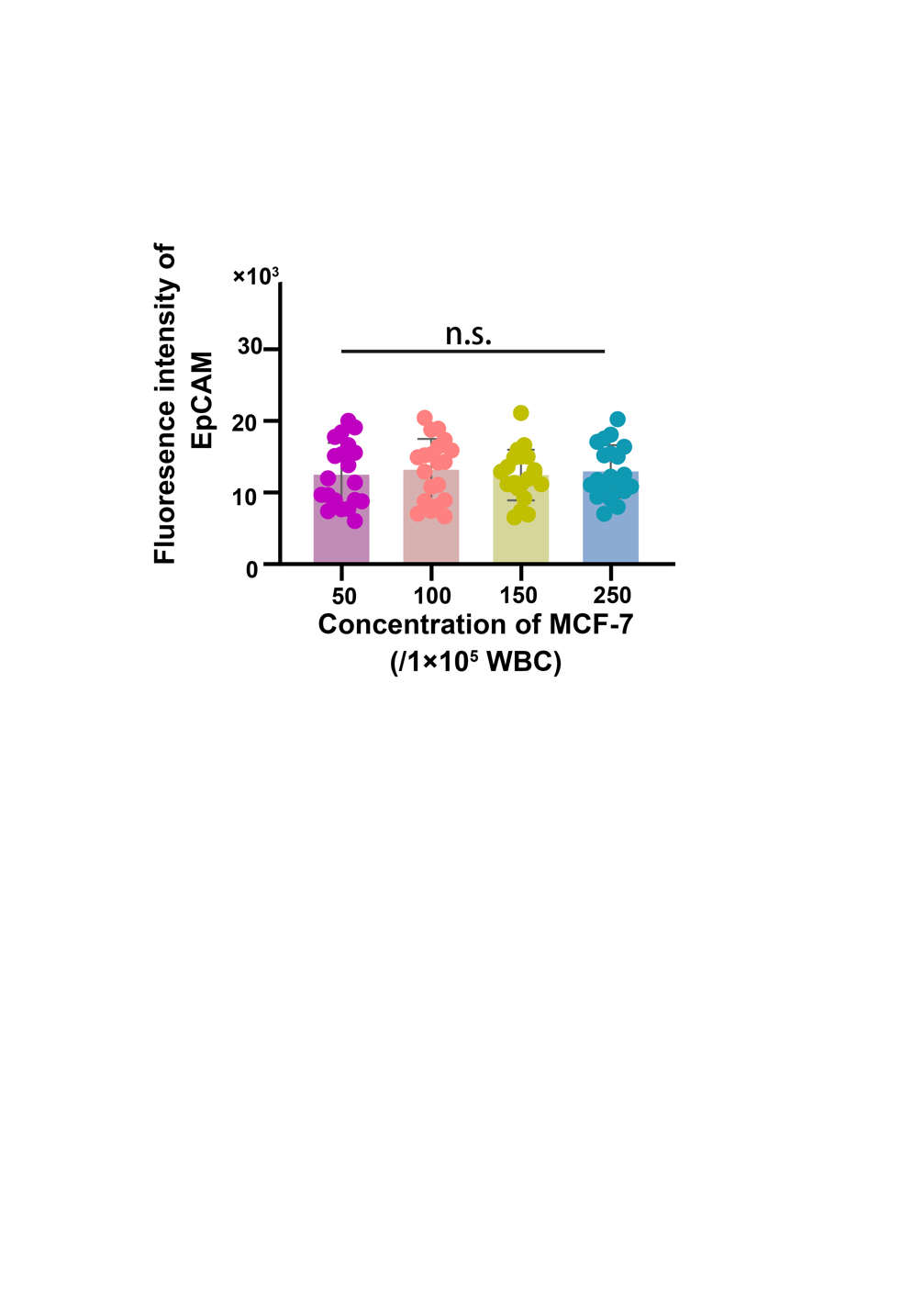


**Figure S7.** Expression profiles of individual MCF-7 cells based on expression of EpCAM via scWB analyses. Data is presented as mean ± SD, where n = 20. n.s. indicates not significant.
